# Endothelial cell senescence is associated with disrupted cell-cell junctions and increased monolayer permeability

**DOI:** 10.1186/2045-824X-4-12

**Published:** 2012-08-28

**Authors:** Vincent J D Krouwer, Liesbeth H P Hekking, Miriam Langelaar-Makkinje, Elsa Regan-Klapisz, Jan Andries Post

**Affiliations:** 1 Department of Biomolecular Imaging, Faculty of Science, Institute of Biomembranes, Utrecht University, Padualaan 8, 3584 CH, Utrecht, The Netherlands

**Keywords:** Senescence, Atherosclerosis, Endothelial junctions, Endothelial permeability, cPLA_2_α

## Abstract

**Background:**

Cellular senescence is associated with cellular dysfunction and has been shown to occur *in vivo* in age-related cardiovascular diseases such as atherosclerosis. Atherogenesis is accompanied by intimal accumulation of LDL and increased extravasation of monocytes towards accumulated and oxidized LDL, suggesting an affected barrier function of vascular endothelial cells. Our objective was to study the effect of cellular senescence on the barrier function of non-senescent endothelial cells.

**Methods:**

Human umbilical vein endothelial cells were cultured until senescence. Senescent cells were compared with non-senescent cells and with co-cultures of non-senescent and senescent cells. Adherens junctions and tight junctions were studied. To assess the barrier function of various monolayers, assays to measure permeability for Lucifer Yellow (LY) and horseradish peroxidase (PO) were performed.

**Results:**

The barrier function of monolayers comprising of senescent cells was compromised and coincided with a change in the distribution of junction proteins and a down-regulation of occludin and claudin-5 expression. Furthermore, a decreased expression of occludin and claudin-5 was observed in co-cultures of non-senescent and senescent cells, not only between senescent cells but also along the entire periphery of non-senescent cells lining a senescent cell.

**Conclusions:**

Our findings show that the presence of senescent endothelial cells in a non-senescent monolayer disrupts tight junction morphology of surrounding young cells and increases the permeability of the monolayer for LY and PO.

## Introduction

Cellular senescence -also called replicative senescence- is defined as an irreversible growth arrest occurring in cultured cells after many population doublings, even though nutrients, growth factors and sufficient space are available for cells to divide. Senescent cells are metabolically active and they undergo profound changes in morphology and function. Cellular senescence is associated with alterations in gene- and protein expression [1-8]. Growing evidence indicates that cellular senescence also occurs *in vivo*, although the detection of senescent cells in tissues is based on markers that are not exclusive for the senescent state [3,9]. The concept that cellular senescence contributes to organismal ageing and to age-related pathologies is well-accepted, but still rather speculative [9]. This concept is supported by the increased occurrence of senescent cells with age, and by the presence of senescent cells specifically at sites of age-related pathologies, such as atherosclerosis [10,11].

Endothelial cells form the monolayer lining the luminal surface of the entire vascular system. One of their main functions is to provide a semi-permeable barrier between the blood and the underlying tissues. Two pathways regulate endothelial permeability, namely the transcellular pathway, by which blood components pass through the cell, and the paracellular pathway, by which the components cross the endothelial barrier through intercellular cell-cell junctions. Endothelial adherens junctions comprise the endothelial-specific transmembrane protein Vascular Endothelial (VE)-cadherin, whereas the transmembrane proteins occludin and endothelial-specific claudin-5 are part of the tight junctions [12]. The integrity of endothelial cell-cell junctions is crucial for the maintenance of vascular homeostasis, including the maintenance of the endothelial barrier function. Loss of junctional integrity, leading to increased vascular permeability, is associated with many pathological disorders [13]. Endothelial cells, among many other cell types, can undergo replicative senescence *in vitro*. Several genomics and proteomics studies on endothelial cell senescence reported changes in gene and protein expression [1,2,7,14]. Some of these changes, such as decreased eNOS activity and decreased NO production [11,15], reduction in mitochondrial membrane potential [5] and increase of ICAM-1 levels [11,16], directly correlate with endothelial dysfunction. Although in general *in vivo* endothelial turnover is low [17], in atherosclerotic-prone areas [18,19] -at bifurcations or other areas of vascular transition- the endothelial cell turnover is expected to be increased because of chronic injury due to changes in shear. Senescent endothelial cells have been identified *in vivo*[20], specifically at sites of atherosclerotic lesions [10,11]. Senescent endothelial cells present at the surface of those lesions express lower levels of eNOS. It has been proposed that senescence of endothelial cells contributes to the onset and/or the progression of atherosclerosis [11,20,21], although a causal relationship has not been proven.

The accumulation of LDL in the intima of the vessel wall is one of the initial steps of atherosclerosis [18,19], this suggests that the endothelium undergoes changes that compromise its barrier function. Endothelial permeability is affected and regulated by many factors. One of the key elements in controlling endothelial permeability is the junctional complex between adjacent endothelial cells. Little is known about the consequences of the presence of senescent endothelial cells on the barrier function of the endothelial monolayer. Recently, a down-regulation in junction protein expression was reported upon inhibition of telomerase activity [22] and notch-induced senescence [23]. In addition, a genomics study indicated a slight down-regulation of claudin-5 in senescent cells [24]. We hypothesize that the presence of replicative senescent cells in an endothelial monolayer affects cell-cell interactions and thereby endothelial permeability. Therefore, in the present work, we set out to determine the integrity of adherens and tight junctions in non-senescent, senescent and in co-cultures of non-senescent and senescent primary human endothelial cells. Furthermore, we examined whether the presence of senescent endothelial cells is associated with increased endothelial permeability.

We recently showed that cytosolic phospholipase A_2_α (cPLA_2_α) is a critical protein regarding the formation and maintenance of tight junctions [25]. Because senescence is accompanied by altered gene expression and cPLA_2_α plays a key role in tight junction regulation, we specifically investigated the expression of this protein in relation to senescence.

## Methods

All data presented in this paper were the result of at least three independent cell isolations and experiments.

### Endothelial cell isolations and cultures

Umbilical cords were obtained from the Department of Obstetrics and Gynecology, Diakonessen Hospital, Utrecht, The Netherlands, with the informed consent of the parents. Human umbilical vein endothelial cells (HUVECs) were isolated and cultured as previous described [1,26,27]. For non-senescent cells, passages 1 to 4 were used; cells were seeded at a density of 10 000 cells/cm^2^ and grown for 7 days, after which they formed a confluent monolayer. Senescent HUVECs were obtained by repetitive culturing of the cells, as described in [1]. Senescent cells were used when cells showed the following senescent characteristics: they seized to proliferate for 3 weeks in complete culture medium and 60%–70% of the cells were positive for SA-β-Gal and γH2AX. These characteristics showed between passage 21 and 28, depending on the HUVEC isolation. Senescent cell cultures were seeded at a density of 70.000 cells/cm^2^; a density more than sufficient to form a confluent monolayer, after which they were maintained in culture for 7 days in order to allow ample time for the formation of adherens and tight junctions.

For all co-culture experiments, non-senescent cells were co-cultured with senescent cells of the same isolation in a ratio of 2:1. To be able to use HUVECs of the same isolation, one part of the HUVECs were directly frozen after isolation, whereas the other part was cultured until senescence. Prior to the experiments, frozen HUVECs were thawed and cultured. The co-culture was allowed to form a confluent layer for 7 days. During these 7 days only the non-senescent cells divide, altering the ratio of non-senescent:senescent cells. Frozen and thawed cells behaved identical to non-frozen cells.

### Antibodies

Mouse anti-VE-cadherin clone TEA 1/31 (1:100 dilution for IF and 1:1000 for WB) was purchased from Immunotech (Marseille, France; Cat. No. 1597). Rabbit anti-occludin (1:100 for IF and 1:1000 for WB, Cat. No. 71-1500) and Mouse anti-claudin-5 (1:200 for IF and 1:1000 for WB, clone 4C3C2, Cat. No. 35-2500) were purchased from Zymed Laboratories (South San Francisco, CA). Mouse anti-cPLA_2_α (1:100 for WB, Cat. No. sc-454) and goat anti-cPLA_2_α (1:30 for IF, Cat. No. sc-1724) were purchased from Santa Cruz Biotechnology (Santa Cruz, CA). Alexa Fluor 488-labeled donkey anti-goat, goat anti-mouse, goat anti-rabbit and Alexa Fluor 555-labeled goat anti-mouse antibodies were purchased from Molecular Probes (Eugene, OR) and all used in 1:500 dilution for IF. Peroxidase-conjugated donkey anti-mouse and donkey anti-rabbit antibodies were purchased from Jackson Immuno Research and used in 1:5000 dilution for WB.

### Immunofluorescence microscopy

Processing of HUVEC samples for immunofluorescence and fluorescent imaging was performed as follows. Cells were fixed for 15 min at RT in 4% PFA diluted in PBS, washed 3 × 5 min, incubated in 0.1% Triton X-100 (diluted in PBS) for 10 min at RT, washed 3 × 2 min and then incubated in 50 mM Glycine in PBS for 10 min at RT. After washing 3 × 5 min, the 12 mm coverslips were incubated on 30 ul drops of primary antibody solution (containing 1% BSA in PBS) for 1 hour at RT. Samples where then washed 3 × 10 min, after which secondary antibodies were incubated in the same manner. After antibody incubation, coverslips where washed for 3 × 10 min, incubated for 5 mins in PBS containing 2 ug/ml DAPI, washed 2 × 5 min in PBS and 1× quickly in H_2_O, and mounted on 4 ul drops of ProLong Gold antifade mounting reagent (Invitrogen, P36930). Samples were allowed to cure at RT for at least 16 hours before microscopical analysis.

The fluorescence intensity of claudin-5 labeling was determined at the cell-cell contacts between 1) a non-senescent and senescent endothelial cell; 2) two non-senescent cells lining a senescent cell; 3) a non-senescent cell lining a senescent cell with a non-senescent cell not directly neighboring a senescent cell and 4) two non-senescent cells not directly neighboring a non-senescent cell. Five images were analyzed using ImageJ software v1.41. In each image, at various locations, 40 lines of 7 μm were drawn perpendicular to the cell-cell contacts. A plot profile was made of the claudin-5 intensity along each line and an area of 2.5 μm around the maximal intensity at the cell-cell contacts was analyzed to determine the fluorescent intensity at the cell-cell contacts. Statistical analysis was performed using ANOVA followed by Tukey’s multiple comparison test.

### Western blot analysis

Seven day confluent non-senescent and senescent HUVECs were washed once with HBSS (PAA Laboratories, Linz, Austria) and incubated on ice for 5 minutes with lysis buffer (25 mM Tris HCl, pH 8, 1% TX100, 100 mM NaCl, 10 mM EDTA, 1x Complete EDTA-free protease inhibitor cocktail (Roche, 11873580001), and 1 mM Na3VO4). Cells were scraped and centrifuged for 10 min at 13,000 rpm at 4°C. Supernatants were collected and protein determination was performed using a BCA protein assay kit (Pierce, Rockford, IL).

Western blot analysis was performed as described previously [28]. Lysate containing 5 μg of total protein was loaded on 12% SDS-PAGE for the detection of claudin-5, and 15 μg total protein on 8% SDS-PAGE for the detection of other proteins. For immuno-detection of cPLA_2_α, the mAb (sc-454) was used.

For quantification, regions of interest of a fixed surface area of at least 3 independent experiments were selected and signal intensities were measured using ImageJ software v1.41. A region of interest right above or below the band of interest was used as background and subtracted from the signal intensity of the band of interest. Bands of interest were normalized by dividing the signal intensity of the band of interest by the signal intensity of the corresponding tubulin band, which was used as a loading control. Signal intensities from different independent experiments were then normalized to each other by expressing the signal fractions of senescent samples as a percentage of non-senescent cells. Statistical significance was determined using paired t-tests.

### Permeability measurements

HUVECs were cultured for 7 days on 0.4 μm pore size PET track-etched membrane transwell filters (BD Biosciences cat. No. 353180) coated with a 1:100 dilution of Matrigel™ (BD Biosciences), see [27] for details. Lucifer Yellow (LY, 20 μg/ml) and peroxidase (PO, 1.2 μg/ml) were added to the upper chamber of the transwells. Samples were taken from the bottom chamber at 1, 2, 3, 4.5, 6 and 24 hours and assayed for fluorescence (LY) or chemoluminescence (PO) using a BMG Fluostar Optima. Data were normalized to the signal obtained for the non-senescent monolayer and statistical analysis was performed using unequal variance ANOVA.

## Results

### HUVECs cultured to passage 28 display characteristics of senescence

**Figure 1:**
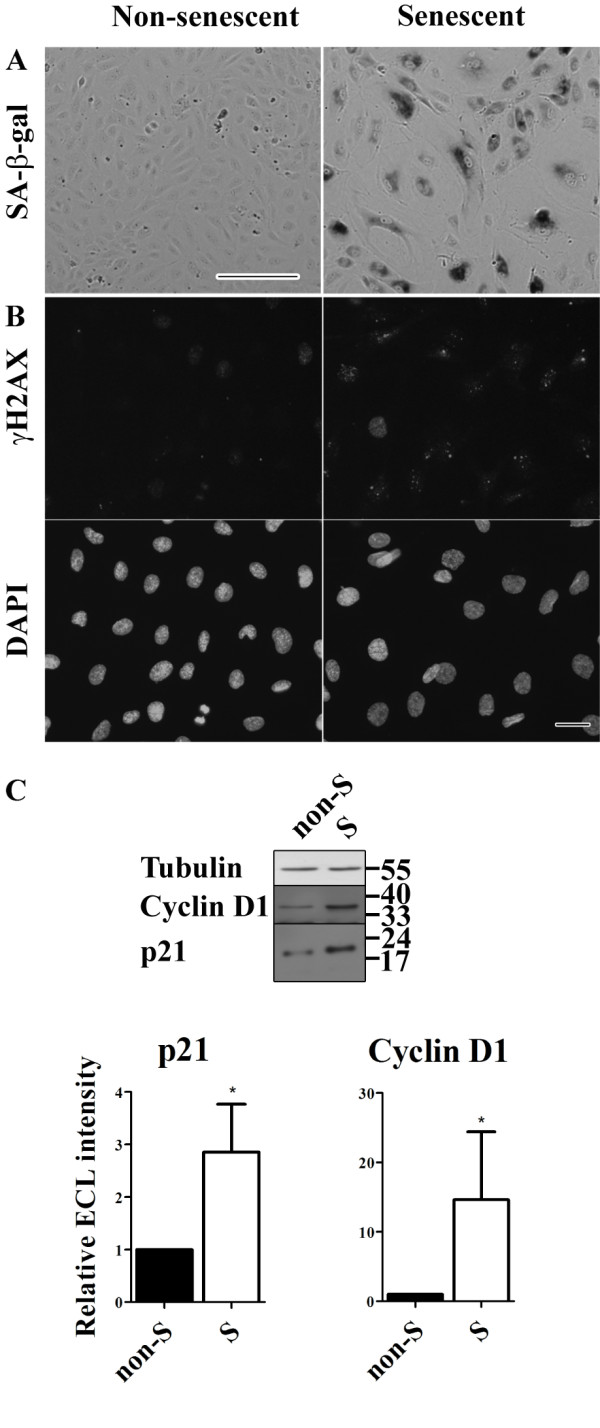
**Characteristics of replicative senescent HUVECs.** (**a**) Senescent-associated β-galactosidase (SA-β-gal) activity staining of non-senescent and senescent HUVECs (bar 250 μm). (**b**) Immunofluorescent labeling of γH2AX with below the corresponding DAPI images to visualize the nuclei of all HUVECs (bar 25 μm). (**c**) Western blot analysis of Cyclin D1 and p21 expression levels in cell lysates of non-senescent and senescent HUVECs. Cyclin D1 and p21 are typical senescence markers. Tubulin was used as a loading control.

We used senescent cells to examine the potential effects of endothelial cell senescence on the integrity of endothelial adherens and tight junctions. The major criterion for cellular senescence was that cells seized to proliferate for 3 weeks in the presence of complete culture medium. About 60%–70% of the cells were positive for senescence-associated β-galactosidase (SA-β-Gal) and γH2AX (Figure 1a & b). None of the senescent cells showed incorporation of 5-ethynyl-2^′^-deoxyuridine (EdU, to monitor DNA replication and thus cell proliferation; data not shown). In addition, senescent cells exhibited a strong increase in expression of classical senescent markers such as p21 and cyclin D1 as determined by Western Blot analysis (Figure 1c).

### Adherens and tight junctions are disrupted in replicative senescent HUVECs

**Figure 2:**
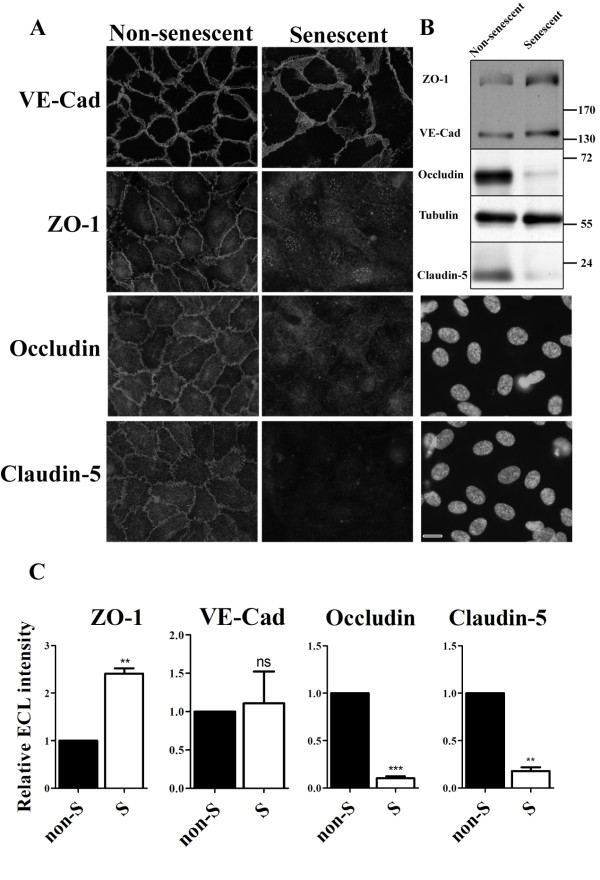
**Junctions are disrupted in replicative senescent HUVECs.** (**a**) Immunofluorescent labeling of junction proteins in senescent and non-senescent HUVECs. The corresponding DAPI staining of senescent HUVECs labeled for occludin and claudin-5 are shown (bar 25 μm). (**b**) Western blot analysis of junction protein expression levels in non-senescent and senescent HUVECs. Tubulin was used as a loading control. (**c**) Quantitative analysis of Western Blots of three independent experiments as performed in (**b**).

To determine the integrity of junctons in non-senescent and senescent HUVEC cultures, we examined the sub-cellular localization of the adherens junction protein VE-cadherin, and of the tight junction proteins ZO-1, occludin and claudin-5. Since senescent cells have lost the capacity to divide, they need to be seeded at high density to be able to form a confluent monolayer with established cell-cell contacts. Therefore, senescent cells were seeded at a higher density than non-senescent cells. Non-senescent or senescent HUVECs were cultured for 7 days, after which the non-senescent HUVECs had formed a confluent monolayer displaying well developed adherens and tight junctions as monitored by immunofluorescence of endogenous VE-cadherin, ZO-1, occludin and claudin-5 (Figure 2a, non-senescent cells). In senescent cells, VE-cadherin was clearly expressed; indicating that the senescent HUVECs indeed establish cell-cell contacts and retain an essential characteristic of the endothelial phenotype. However, the distribution pattern of VE-cadherin labeling at the cell-cell contacts was different and the adherens junctions appeared swollen en disorganized (Figure 2a, senescent cells). Of the tight junction proteins, ZO-1 was clearly detectable in senescent cells but the distribution of the labeling was different: at cell-cell contacts the distribution was very faint. Instead, ZO-1 appeared to be located more to the cytosol compared to non-senescent cells. Western Blot analysis confirmed normal expression levels of VE-cadherin and ZO-1 in senescent cells (Figure 2b & c).

In contrast to the relatively small differences in VE-cadherin and ZO-1 distribution and expression, huge differences were observed for tight junction proteins occludin and claudin-5: whereas in non-senescent HUVECs occludin and claudin-5 were expressed and located at cell-cell contacts, very little labeling was observed in senescent cells (Figure 2a). Western-blot analysis indeed showed strong down-regulation in expression levels of occludin and claudin-5 in senescent HUVECs (Figure 2b & c).

### Adherens and tight junctions are disrupted between replicative senescent HUVECs and young HUVECs

**Figure 3:**
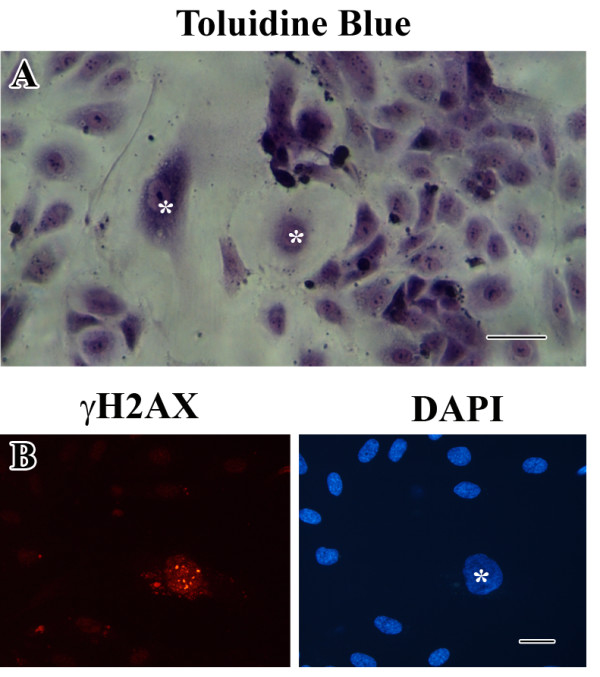
**Co-culture of young and senescent HUVECs.** (**a**) Representative photograph of toluidine blue staining of a co-culture (bar 100 μm). (**b**) Immunofluorescent labeling of γH2AX. Only the senescent HUVEC displays positive labeling for γH2AX. Right image shows the corresponding DAPI staining (bar 25 μm).

When endothelial cells become senescent *in vivo *[10,11,20], they are most likely in contact with neighboring non-senescent endothelial cells. To mimic this situation, we decided to co-culture non-senescent and senescent HUVECs originating from the same umbilical cord (Figure 3).

**Figure 4:**
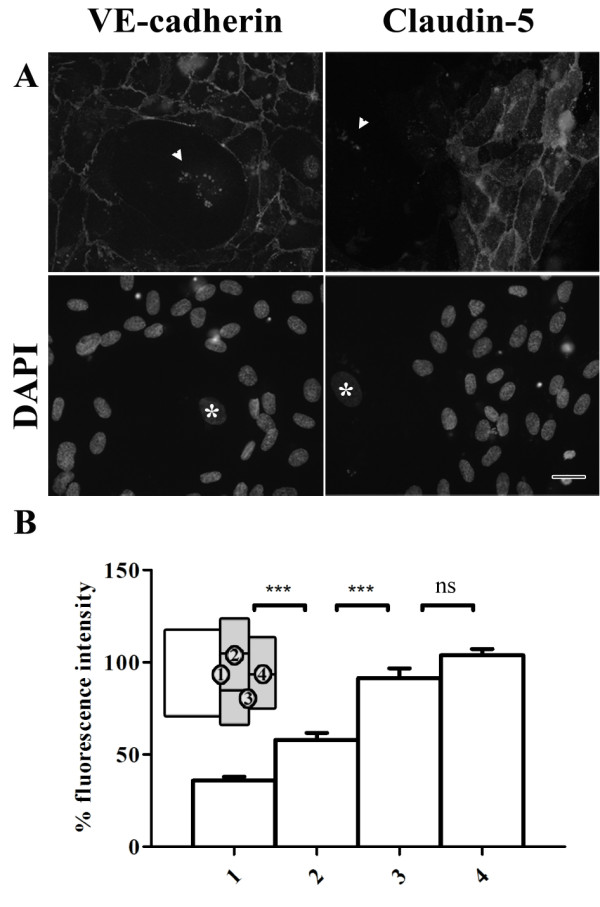
**Cell-cell contacts between young and senescent HUVECs are disrupted in co-cultures.** (**a**) Immunofluorescent labeling of VE-cadherin and claudin-5 in co-cultures of senescent and non-senescent HUVECs (bar 25 μm). Bottom row shows the corresponding DAPI labeling. The white arrows in the upper row indicate lipofuscin autofluorescence; the white stars in the lower row indicate the nucleus of senescent cells. (**b**) Fluorescence intensity of claudin-5 at cell-cell contacts, normalized against the mean intensity at position 4. Numbers at the x-axis correspond to the positions indicated in the schematic representation (inset) showing the senescent cell in white and the non-senescent cells in gray.

Normal distributions of adherens junctions and tight junctions were observed in regions with non-senescent HUVECs (Figure 4a). However, at the cell-cell contacts between senescent cells and non-senescent cells the labeling pattern of VE-cadherin was less regular. In agreement with the distribution of claudin-5 at cell-cell contacts of senescent cells shown in Figure 2, no expression of claudin-5 was observed between senescent cells.

### Tight junctions are disrupted between young HUVECs that are in the vicinity of a senescent HUVEC

Intriguingly, non-senescent HUVECs lining a senescent HUVEC displayed reduced expression of claudin-5 along its entire periphery, even at cell-cell contacts with neighboring non-senescent cells. This was confirmed by quantification of the labeling intensity of claudin-5 at the cell-cell contacts between 1) a non-senescent and senescent cell; 2) two non-senescent cells lining a senescent cell; 3) a non-senescent cell lining a senescent cell with a non-senescent cell not directly neighboring a senescent cell and 4) two non-senescent cells not directly next to a non-senescent cell (Figure 4b). Significant lower fluorescence intensity was found at the cell-cell contacts between a senescent cell and a non-senescent cell compared to the intensity found between two non-senescent cells.

### Co-cultures of young and senescent HUVECs display increased permeability for PO and LY

**Figure 5:**
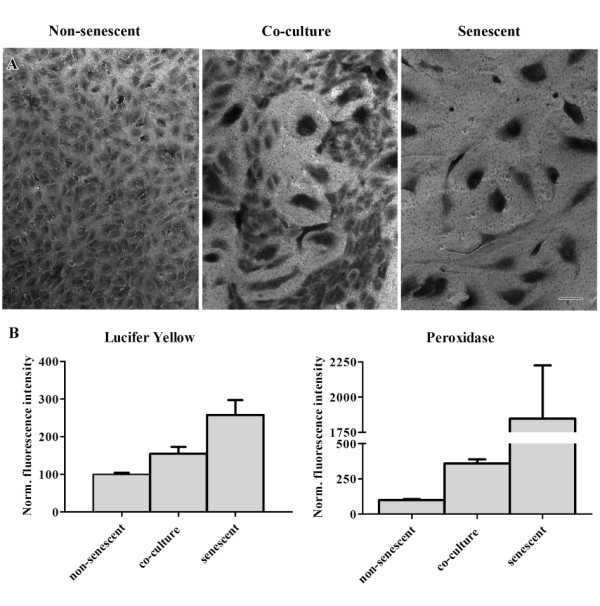
**Senescence is associated with increased endothelial permeability.** (**A**) Representative light microscopy images of non-senescent, co-cultured and senescent HUVECs on transwell filters (bar 100 μm). The cells were fixed after the permeability assay was performed and stained with toluidine blue. (**B**) Normalized values of transport through cultured non-senescent, co-culture or senescent HUVECs one hour after addition of LY or 24 hours after addition of PO. The values are normalized against the mean value of the non-senescent cultures. p < 0.01 in all cases.

The observed alterations in distribution and expression of junction proteins at cell-cell contacts in non-senescent vs. senescent HUVECs and in co-cultures might affect the barrier function of the aged endothelial monolayer. By using the transwell system we studied over time the permeability for lucifer yellow (LY; mw = 450 Da) and horseradish peroxidase (PO; mw = 44000 Da) across the various types of monolayers. The earliest time-point was 1 hour after the addition of the tracer molecules; later samples were taken at 2, 3, 4.5, 6 and 24 hours. Microscopical analysis of the filters clearly showed that confluent cell-layers were present at the time of the permeability assay (Figure 5A).

Already one hour after the start of the assay, 2.5 times more LY had passed through the senescent monolayer compared to the non-senescent monolayer (Figure 5B, Lucifer Yellow). In the co-cultures, the amount of LY that had passed through the monolayer was 1.5× higher compared to non-senescent cell cultures.

For the high molecular weight tracer, PO, the senescent cell cultures also displayed highest permeability. Differences in the passage of PO over the different cultures were most pronounced at the later time-points 6 and 24 hours; 24 hours after the addition of the marker, PO activity was found to be 18× higher in the lower compartments of the transwells seeded with senescent HUVECs compared to the non-senescent HUVECs (Figure 5b, Peroxidase). In comparison, at the same time-point the lower compartments of the co-cultures displayed a 3.5× higher PO activity compared to non-senescent HUVECs.

### cPLA_2_α expression is down-regulated upon senescence

In a recent study, we showed that cPLA_2_α is a key player in the formation and maintenance of endothelial cell-cell junctions [25]. Silencing of cPLA_2_α or inhibition of cPLA_2_α activity resulted in an altered distribution of adherens junctions and reduced presence of tight junction proteins at the cell-cell contacts of cultured HUVECs [25]. Since we observe a similar effect on adherens and tight junctions upon senescence of cultured HUVECs, we decided to study the expression of cPLA_2_α in these cells.

**Figure 6:**
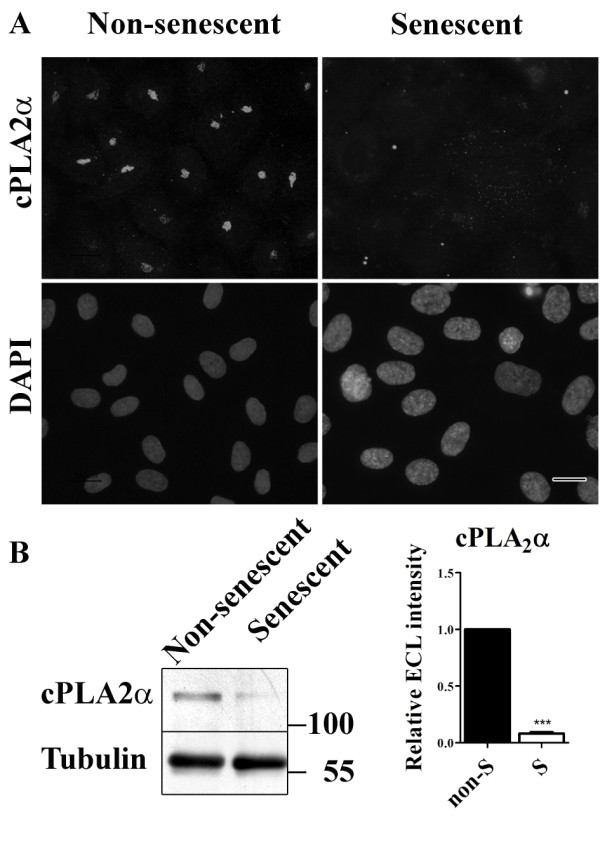
**cPLA**_**2**_**α is down-regulated in senescent HUVECs.** (**a**) Immunofluorescent labeling of cPLA_2_α in non-senescent and senescent HUVECs (bar 25 μm). (**b**) Western blot analysis to detect cPLA_2_α in lysates of non-senescent and senescent HUVECs. Tubulin was used as a loading control.

In confluent, non-senescent HUVECs, cPLA_2_α was clearly present and located at the Golgi as previously described [25] (Figure 6a). In senescent HUVECs however, a much weaker labeling pattern of cPLA_2_α was observed. Western blot analysis shows that this is at least partly caused by reduced expression of cPLA_2_α (Figure 6b).

## Discussion

In this study we provide evidence that replicative senescence causes disrupted cell-cell contacts between endothelial cells (HUVECs). These disrupted cell-cell contacts are a result of alterations in the distribution of adherens junction proteins and decreased expression of tight junction proteins. These alterations are not only observed between senescent cells, but also at cell-cell contacts between non-senescent and senescent cells and along the entire periphery of non-senescent cells lining a senescent cell. The alterations in distribution and expression of junction proteins coincides with increased permeability for small (450 Da) and large (44 kDa) tracer molecules, indicating that the barrier function is compromised. Monolayers of co-cultures of non-senescent and senescent HUVECs also showed increased permeability for both tracer molecules. In addition, the expression of cPLA_2_α, that has been shown to play a role in the maintenance of the integrity of the endothelial barrier [25], was severely down-regulated in senescent cells. This down-regulation of cPLA_2_α might play a role in the decreased barrier function of senescent monolayers.

Senescent endothelial cells have been identified at sites of atherosclerotic lesions [10,11] and have been proposed to contribute to the onset and/or the progression of atherosclerosis [11,20,21]. However, a causal relationship between senescence and the development of atherosclerosis has not yet been proven and, to our knowledge, the effect of replicative senescence on endothelial cell-cell contacts has not been studied before. Using non-replicative senescence models (either over-expression or silencing of specific proteins) did indicate altered barrier function of an endothelial monolayer [22,23]. Venkatesh *et al*. [23] observed that the activation of the notch signaling pathway in endothelial cells results in a senescence-like phenotype and is associated with increased permeability of the monolayer caused by altered VE-cadherin and beta-catenin expression and localization. Huang *et al.*[22] showed that in human brain endothelial cells, silencing of TERT or inhibition of TERT activity, leads to senescent features and affects the junctional complexes, which is accompanied by reduced expression of tight junction proteins, including ZO-1. However, no functional measurements were done is this study. In the present study we did not observe down-regulation of ZO-1, but we did observe an alteration in distribution of ZO-1.

We have recently shown that Golgi-localized cPLA_2_α is involved in Golgi-to-plasma membrane trafficking of junction proteins [25]. The down-regulation of cPLA_2_α in senescent cells, as described in this paper, could therefore disrupt the trafficking of junction proteins to the plasma membrane and thereby altering the distribution and expression of these proteins. Taddei *et al.*[29] reported that in endothelial cells clustering of VE-cadherin at the cell-cell junctions is necessary to induce transcription of claudin-5. We hypothesize that disrupted trafficking of VE-cadherin to the plasma membrane of senescent cells, at least partly due to the decreased expression of cPLA_2_α, causes a decrease in VE-cadherin signaling in surrounding young cells. This subsequently leads to decreased expression of claudin-5 in these young cells.

Senescent cells are known to secrete factors that can affect the structure and function of neighboring cells [30]. However, the fact that the effect of senescent cells on junction morphology is only observed in cells that are in contact with senescent cells suggests that, in our setup, secreted factors do not play a role. This is supported by our finding that pre-conditioned medium from senescent cells did not effect on the distribution pattern of claudin-5 and VE-cadherin in non-senescent cells (data not shown).

We hypothesize that, *in vivo,* the presence of senescent endothelial cells exerts the same effect on endothelial barrier function as in the current *in vitro* model. Accumulation of low density lipoprotein (LDL) in the intima can be, at least partially, attributed to disrupted endothelial junctions [31] and is seen as one of the initial steps in atherogenesis [18,19], especially at bifurcations where blood flow is disturbed. Since disturbed flow increases endothelial cell turnover *in vivo *[32,33], it is to be expected that endothelial cells at these locations senesce faster. Indeed, senescent cells have been identified *in vivo* in atherosclerotic tissue [10,11]. Once the atherosclerotic process starts, the extensive oxidation of LDL in the intima could lead to mitochondrial damage and ROS production in endothelial cells [34], further promoting cellular senescence directly or indirectly by an increased cell death and thus increased cell turnover. Mathematical modeling of the dynamics of endothelial cell damage, repair and telomere shortening suggests that in humans, at an age of 65 years, approximately 2-5% of the vascular endothelial cells are senescent [17].

The presence of senescent endothelial cells might affect the atherosclerotic process in several ways. First of all, the compromised junctional complexes and the subsequent increase in vascular permeability due to the presence of senescent cells *in vivo* might increase the transport of LDL over the endothelium, as described above. Moreover, endothelial cell senescence is accompanied by an increase in adhesive properties towards macrophages [7,16]. This, in combination with the described decrease in tight junction protein expression upon senescence, might aid infiltration of the macrophages into the vessel wall, especially since claudin-5 is thought to be one of the most important junction proteins in permeability control [29].

## Conclusion

In this study we provide evidence for the detrimental effect of the presence of senescent endothelial cells in a non-senescent endothelial monolayer. Replicative senescence affects the adherens junctions and, even more strongly, tight junctions, and compromises the integrity of the endothelial barrier. Endothelial cell senescence is accompanied by a down-regulation of cPLA_2_α and this down-regulation might be involved in the described alterations in cellular junctions. It is tempting to speculate that *in vivo* the presence of senescent endothelial cells also results in decreased endothelial barrier function and thereby plays an important role in the initiation and propagation of atherosclerosis. This clearly needs further validation.

## Abbreviations

cPLA_2_α: Cytosolic phospholipase A_2_ alpha; EdU: 5-ethynyl-2^′^-deoxyuridine; eNOS: Endothelial nitric oxide synthase; TERT: Telomerase reverse transcriptase; HUVEC: Human umbilical vein endothelial cell; ICAM-1: Intercellular adhesion molecule 1; LDL: Low density lipoporotein; LY: Lucifer yellow; NO: Nitric oxide; PO: Peroxidase; ROS: Reactive oxygen species; SA-β-Gal: Senescence associated β-galactosidase; VE: Vascular endothelial; ZO-1: Zonula occludens 1.

## Competing interests

The authors declare that they have no competing interests.

## Authors’ contributions

VK, LH, MLM, and ER performed the experiments. VK, ER and JAP designed the experiments, VK, LH and ER wrote the manuscript and JAP supervised the project. All authors read and approved the final manuscript.
